# Herpes Simplex Virus Reactivation and Candida glabrata Fungemia Following Short-Term Corticosteroids in a Patient With Septic Shock

**DOI:** 10.7759/cureus.85851

**Published:** 2025-06-12

**Authors:** Purnoor Kaur, Dhayananth Rattaipalivalasu Saravanan, Manogna Pendyala, Brandon M Wong, Maryam Saghir

**Affiliations:** 1 Internal Medicine, St. Vincent's Medical Center, Toledo, USA

**Keywords:** candida glabrata, drug-induced immunosuppression, high-dose corticosteroids, hsv-1, opportunistic infection, septic shock

## Abstract

Opportunistic infections such as herpes simplex virus type 1 (HSV-1) reactivation and *Candida glabrata* fungemia are traditionally associated with chronic or profound immunosuppression. However, emerging evidence indicates that even brief, high-dose corticosteroid therapy can transiently impair immune function and predispose critically ill patients to severe secondary infections. We present a case of a 52-year-old man with multiple comorbidities who developed HSV-1 dermatitis with gingivostomatitis and *C. glabrata* fungemia following a short course of corticosteroids administered for septic shock. The patient's HSV-1 dermatitis was treated with acyclovir, and *C. glabrata* fungemia was treated with micafungin. This dual infectious complication occurred despite the absence of prolonged immunosuppressive therapy or invasive mechanical ventilation. The case underscores the underrecognized immunosuppressive potential of short-term corticosteroids in the intensive care unit (ICU). It highlights the need for heightened clinical vigilance, especially in patients with predisposing factors such as diabetes, renal impairment, and recent surgical instrumentation.

## Introduction

Systemic corticosteroids are widely used in managing vasopressor-refractory septic shock because they modulate the host inflammatory response and improve hemodynamic stability. The Surviving Sepsis Campaign recommends intravenous (IV) hydrocortisone at doses of up to 200 mg/day in patients who cannot maintain their blood pressure adequately despite fluid therapy and vasopressors [1]. While the long-term immunosuppressive consequences of corticosteroid therapy are well recognized, growing evidence suggests that even brief, high-dose courses can compromise immune function and increase susceptibility to secondary infections [2,3]. These effects become particularly significant in critically ill patients, who face multiple factors that compromise their immune defenses. Their comorbidities reduce their ability to fight infections, while invasive procedures such as ventilation and catheterization compromise natural barriers. Recent studies have demonstrated that short-term corticosteroid use can significantly elevate the risk of opportunistic infections [4]. Herpes simplex virus type 1 (HSV-1) is a DNA virus that remains latent in the sensory ganglia after the initial infection. It may reactivate in the setting of immunosuppression [5]. *Candida glabrata* has become an increasingly important cause of fungemia in intensive care unit (ICU) patients, driven by its intrinsic resistance to azole antifungals and strong association with prior broad-spectrum antibiotic use [6].

In this report, we describe a critically ill patient who developed HSV-1 dermatitis with gingivostomatitis and *C. glabrata* fungemia after a short course of corticosteroids for septic shock. The occurrence of HSV-1 dermatitis followed by *Candida glabrata* infections is unusual after a short course of steroids in the absence of chronic immunosuppression. This case aims to highlight the importance of anticipating opportunistic infections even during limited-duration steroid therapy, particularly in high-risk individuals.

## Case presentation

A 52-year-old man with a medical history of insulin-dependent type 2 diabetes mellitus, stage 3 chronic kidney disease, hypothyroidism (on levothyroxine), and benign prostatic hyperplasia underwent cystoscopy and greenlight photovaporization of the prostate. Three days postoperatively, he presented to the emergency department with fever, chills, hypotension, and tachycardia. Initial laboratory workup (Tables 1-3) revealed a lactate of 4.9 mmol/L, a white blood cell count of 21×10⁹/L, and an elevated creatinine of 2.1 mg/dL (baseline: 1.3-1.4 mg/dL). Urinalysis was suggestive of a urinary tract infection.

**Table 1:** Initial laboratory results This table summarizes the patient's initial routine serum laboratory findings, including renal, hepatic, metabolic, and endocrine panels

Test	Result	Reference Range
Sodium (mmol/L)	137	136-145
Potassium (mmol/L)	4.0	3.7-5.3
Chloride (mmol/L)	104	98-107
Carbon Dioxide (CO₂, mmol/L)	18	20-31
Blood Urea Nitrogen (BUN, mg/dL)	16	6-20
Creatinine (mg/dL)	2.1	0.7-1.2
Anion Gap (mmol/L)	15	9-16
Estimated Glomerular Filtration Rate (eGFR, mL/minute/1.73 m²)	37	>60
Lactic Acid (mmol/L)	4.9	0.5-1.9
Glucose (mg/dL)	192	74-99
Calcium (mg/dL)	9.3	8.6-10.4
Albumin/Globulin Ratio	1.0	1.0-2.5
Total Protein (g/dL)	6.9	6.6-8.7
Troponin, High Sensitivity (ng/L)	13	0-22
N-terminal Pro-B-Type Natriuretic Peptide (NT-pro-BNP, pg/mL)	95	0-125
Albumin (g/dL)	3.5	3.5-5.2
Alkaline Phosphatase (U/L)	94	40-129
Alanine Aminotransferase (ALT, U/L)	15	10-50
Aspartate Aminotransferase (AST, U/L)	25	10-50
Total Bilirubin (mg/dL)	0.7	0.0-1.2
Lipase (U/L)	36	13-60
Thyroid-Stimulating Hormone (TSH, uIU/mL)	4.11	0.27-4.20

**Table 2:** Venous blood gas and point-of-care (POC) results This table summarizes venous blood gas values and point-of-care results

Test	Result	Reference Range
Venous pH	7.380	7.320-7.430
Venous Partial Pressure of CO₂ (pCO₂, mm Hg)	32.6	41.0-51.0
Venous Partial Pressure of O₂ (pO₂, mm Hg)	22.6	30.0-50.0
Venous Bicarbonate (mmol/L)	19.3	22.0-29.0
Base Excess (mmol/L)	5.1	0.0-2.0
Venous Oxygen Saturation (SvO₂, %)	38.6	60.0-85.0
Point-of-Care Total CO₂ (TCO₂, mmol/L)	19	22-30
Point-of-Care Ionized Calcium (mmol/L)	1.11	1.15-1.33
Point-of-Care Potassium (mmol/L)	3.2	3.5-4.5
Point-of-Care Sodium (mmol/L)	146	138-146
Point-of-Care Hematocrit (%)	30	41-53

**Table 3:** Initial complete blood count This table shows the complete blood counts of the patient on the day of admission

Parameter	Result	Reference Range	Interpretation
White Blood Cell (WBC) Count	21.7	4.0-10.0×10⁹/L	High
Red Blood Cell (RBC) Count	3.82	4.5-5.9×10¹²/L	Low
Hemoglobin	9.5	13.5-17.5 g/dL	Low
Hematocrit	29.7	41%-53%	Low
Mean Corpuscular Volume (MCV)	77.7	80-100 fL	Low
Mean Corpuscular Hemoglobin (MCH)	24.9	27-33 pg	Low
Mean Corpuscular Hemoglobin Concentration (MCHC)	32.0	32-36 g/dL	Normal
Mean Platelet Volume (MPV)	11.2	7.4-10.4 fL	High
Red Cell Distribution Width (RDW)	15.3	11.5%-14.5%	High
Platelet Count	264	150-450×10⁹/L	Normal
Neutrophils, %	92	40%-70%	High
Lymphocytes, %	2	20%-45%	Low
Monocytes, %	2	2%-10%	Normal
Eosinophils, %	1	1%-6%	Normal
Basophils, %	0	<1%	Normal
Absolute Neutrophils	19.97	2.0-7.5×10⁹/L	High
Absolute Lymphocytes	0.43	1.0-3.0×10⁹/L	Low
Absolute Monocytes	0.43	0.2-1.0×10⁹/L	Normal
Absolute Eosinophils	0.22	0.04-0.4×10⁹/L	Normal
Absolute Basophils	0.00	<0.1×10⁹/L	Normal
Immature Granulocytes, %	3	0%-1%	High
Immature Granulocytes, Absolute	0.65	0-0.3×10⁹/L	High
Nucleated Red Blood Cells (NRBC)	0.3	0/100 WBC	High
Morphology	-	-	Anisocytosis and Microcytosis

**Figure 1:**
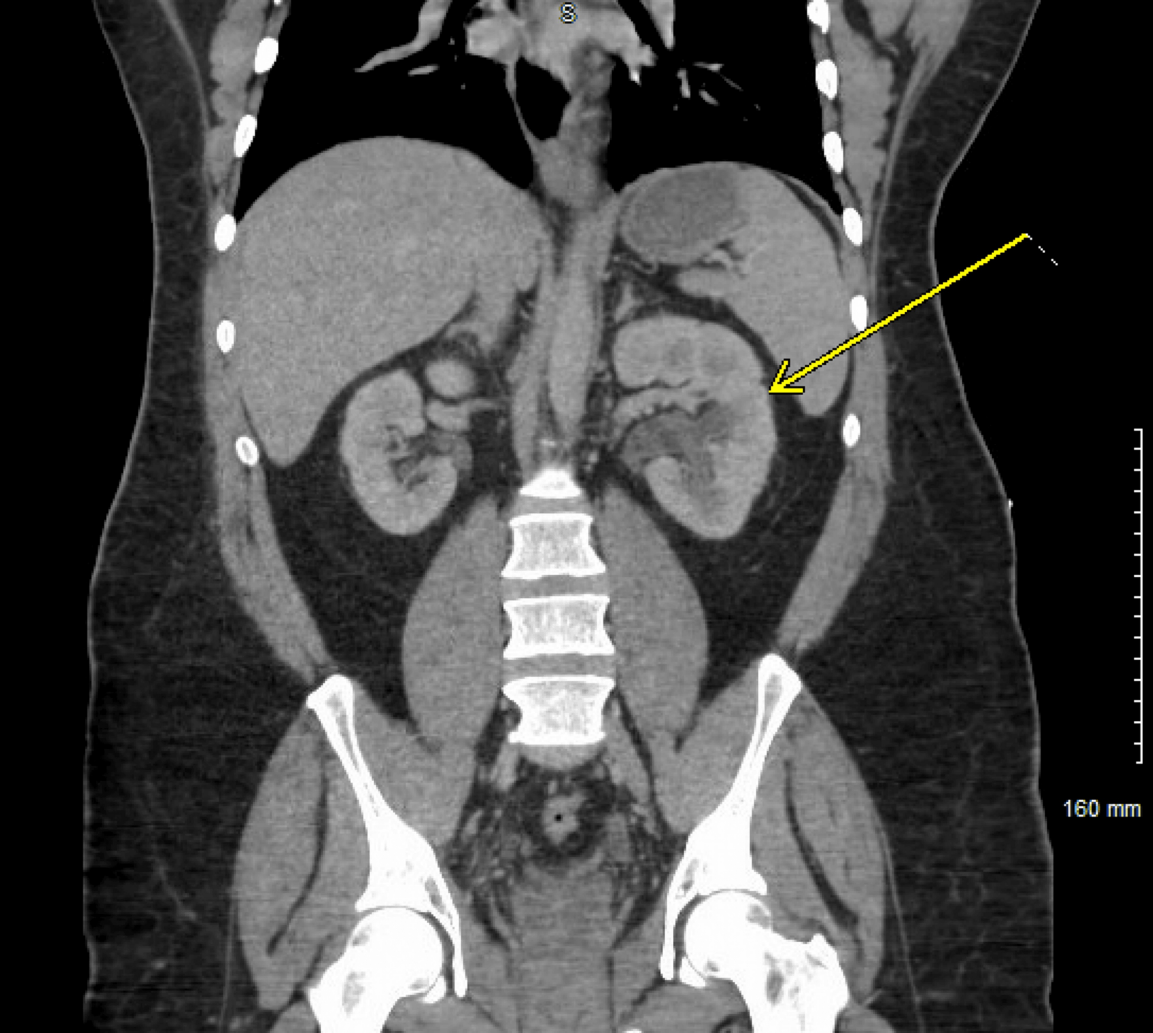
Left hydronephrosis CT scan of the abdomen and pelvis showing mild left hydronephrosis, pointed by a yellow arrow

**Figure 2:**
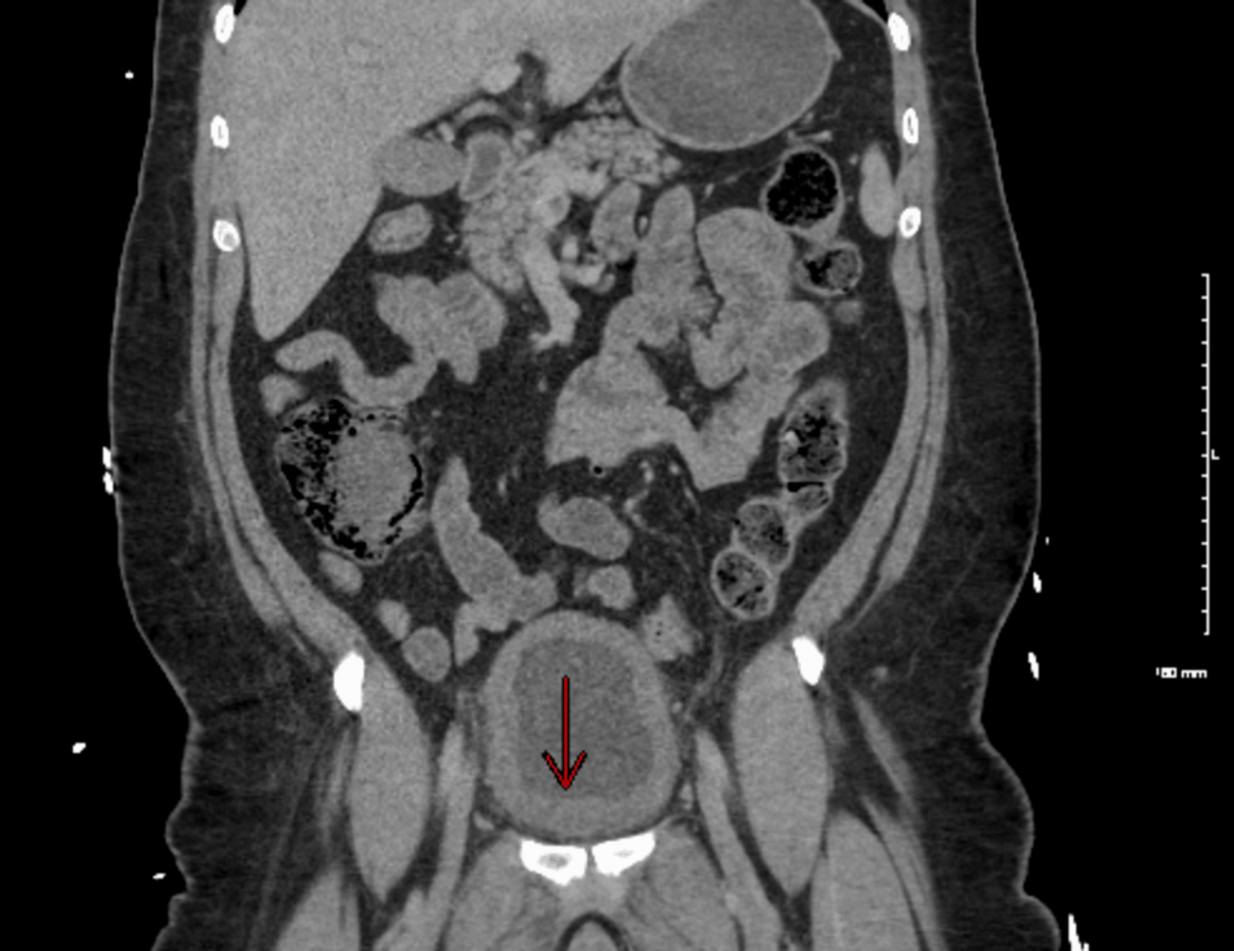
Bladder wall thickness CT scan of the abdomen and pelvis showing increased bladder wall thickness, pointed by a red arrow

**Figure 3:**
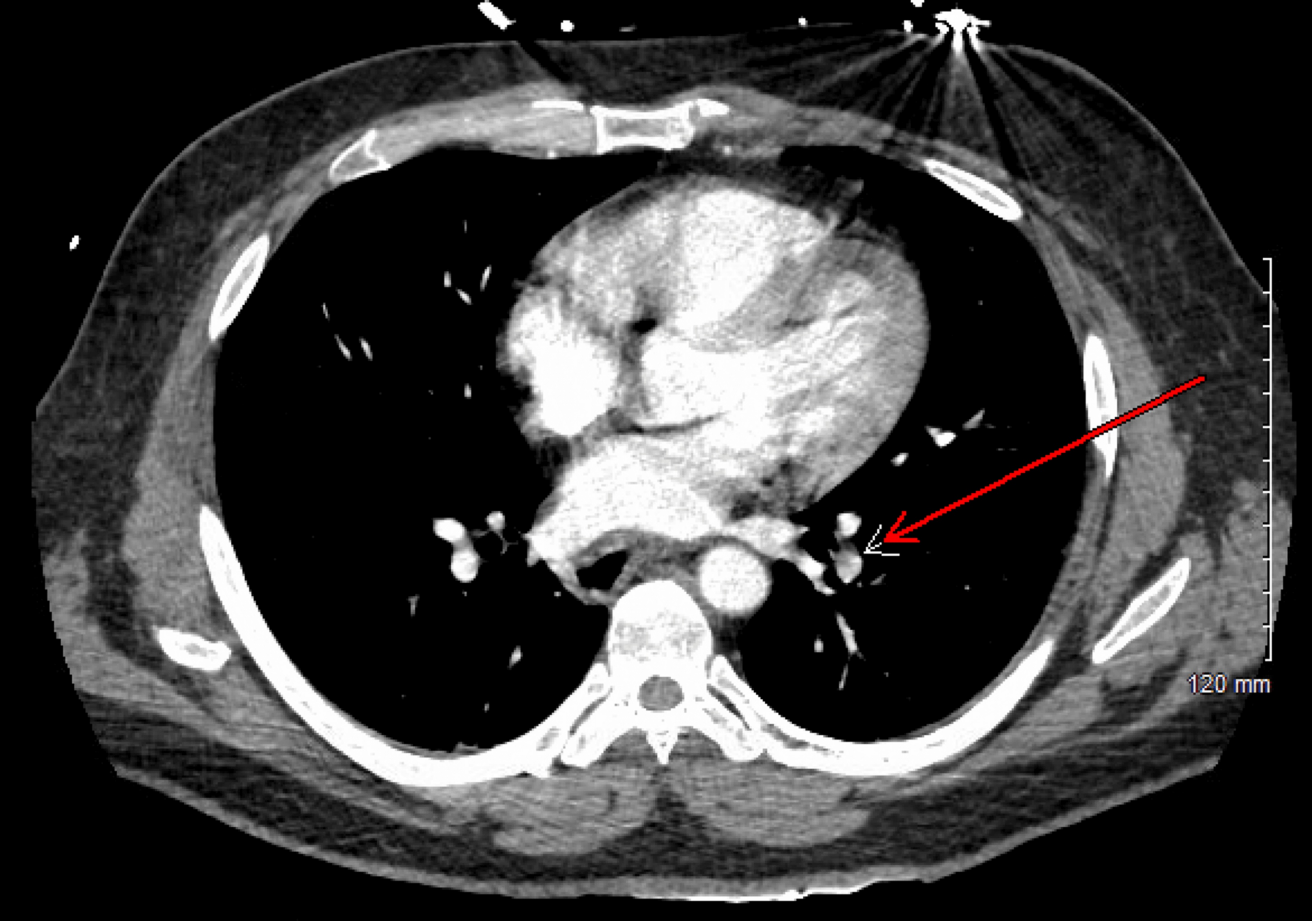
CTPA of the chest showing pulmonary embolism, pointed by a red arrow CTPA: CT pulmonary angiography

CT imaging of the abdomen showed diffuse mild left-sided hydronephrosis (Figure 1) and bladder wall thickness (Figure 2), while CT pulmonary angiography revealed two small nonocclusive pulmonary emboli (Figure 3).

Despite fluid resuscitation, the patient remained hypotensive and was admitted to the intensive care unit (ICU) for septic shock. He was started on norepinephrine, IV fluids, vancomycin, and piperacillin-tazobactam. Stress-dose hydrocortisone (~900 mg over four days) (Table 4) was initiated due to concern for adrenal insufficiency. The patient refused the placement of a central line on admission for vasopressor infusion. Anticoagulation with apixaban (Eliquis) was started for pulmonary embolism, but it had to be discontinued due to gross hematuria and the passage of blood clots in the urine. A lower extremity Doppler ultrasound revealed acute deep vein thrombosis in the left soleal and posterior tibial veins. Heparin infusion was started but had to be discontinued due to persistent hematuria. Vascular surgery advised holding anticoagulation and repeating a duplex study in two to three weeks. They recommended no further anticoagulation if there was no thrombus propagation to the popliteal vein.

**Table 4:** In-hospital steroid administration ICU: intensive care unit

Date	Day 1	Day 2	Day 3	Day 4	Day 5	Day 6
Patient Station	ICU	ICU	ICU	ICU	Medicine Floors	Medicine Floors
Solu-Cortef	200 mg	300 mg	300 mg	100 mg	-	-
Prednisone	-	-	-	-	-	20 mg

Acute kidney injury was attributed to a combination of urinary obstruction, sepsis, and contrast nephropathy. Bicarbonate-containing fluids were administered, and a Foley catheter was inserted on ICU day 2. Blood and urine cultures grew *Serratia marcescens*, which was resistant to cefazolin but sensitive to ceftriaxone and levofloxacin. Antibiotics were de-escalated accordingly, and the patient was transitioned from vancomycin and piperacillin-tazobactam to levofloxacin. A transthoracic echocardiogram showed a preserved ejection fraction with no vegetation. He was weaned off pressor support on day 3. With clinical improvement, his creatinine level returned to baseline, and the patient was transferred to the medical floor under the care of the internal medicine service.

**Figure 4:**
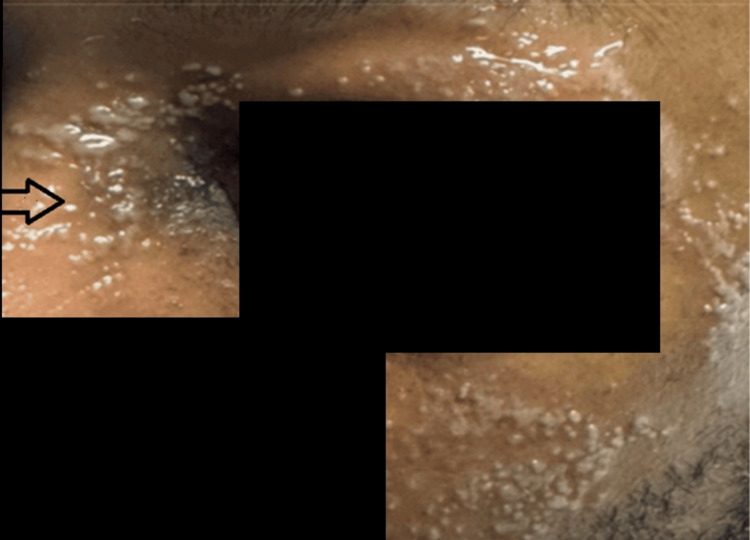
Vesiculopapular rash The image shows a vesiculopapular rash around the V2 zone

On hospital day 6, a new, non-painful rash was noted over the left periorbital region (Figure 4). Dermatology was consulted, and an acute localized exanthematous pustulosis (ALEP) diagnosis was strongly favored. This suggestion was based on case reports and suspected triggers, which include cefazolin (administered intraoperatively), piperacillin-tazobactam, vancomycin, and levofloxacin, in descending order of likelihood. Less likely differential diagnoses included herpes zoster and cellulitis. The patient also developed an acute gout flare with a uric acid level of 7.4 mg/dL. He was started on low-dose prednisone at 20 mg daily; however, this was discontinued after the progression of the facial rash. Infectious disease consultation recommended switching antibiotics to ceftriaxone, which was continued to treat *Serratia marcescens* bacteremia.

By the following day, the rash had evolved into vesicular lesions with mucosal involvement and eye discharge. The patient endorsed a prior history of cold sores, and IV acyclovir was initiated. Ophthalmology diagnosed left V2 HSV-1 dermatitis with gingivostomatitis; no ocular complications were noted. Although polymerase chain reaction (PCR) testing was not performed, the clinical diagnosis was considered sufficient. Acyclovir was transitioned to oral valacyclovir and completed as a five-day antiviral course.

On hospital day 8, the patient developed a new fever (maximum temperature {Tmax}: 103°F). Although the herpetic rash showed improvement, the persistent fever prompted further evaluation. Blood cultures drawn returned positive for *Candida glabrata*. IV micafungin (100 mg daily) was initiated and continued for 14 days following culture clearance. A comprehensive immunodeficiency workup, including an HIV test, hepatitis panel, antinuclear antibodies (ANA), complement levels, and immunoglobulin levels, was unremarkable. The patient had a Foley catheter placed on the day of admission, which could potentially be the source of fungemia.

Midline was placed for the completion of intravenous antibiotics, and he was discharged in stable condition. The patient ultimately completed seven days of IV ceftriaxone for *Serratia marcescens*, five days of antiviral therapy (IV acyclovir transitioned to oral valacyclovir), and 14 days of IV micafungin for *Candida glabrata* fungemia. He followed up with his primary care physician (PCP) two weeks later, who noted the complete resolution of his rash, fever, and fatigue, as well as improvement in his general health.

## Discussion

Corticosteroid use in septic shock remains a double-edged sword. While they play a vital role in the management of vasopressor-refractory septic shock, especially in patients with relative adrenal insufficiency, their immunosuppressive effects can predispose patients to serious infections. Glucocorticoids exert broad immunomodulatory actions by suppressing proinflammatory cytokines (e.g., IL-1, IL-6, and tumor necrosis factor-alpha {TNF-α}), inhibiting antigen presentation, reducing neutrophil chemotaxis, and impairing the phagocytic and autophagic activity of macrophages [1,2]. Though therapeutic in sepsis, these effects may blunt the innate immune response and promote the reactivation or acquisition of opportunistic infections.

Recent data have highlighted that even short courses of corticosteroids may be associated with an increased risk of infection. A population-based study by Waljee et al. found that a median six-day course of oral corticosteroids was associated with a higher risk of sepsis (OR: 5.30), venous thromboembolism (OR: 3.33), and fracture (OR: 1.87) within the first 30 days of therapy [3]. This study mainly compared the risk of complications in steroid users to nonusers, with the median dose of steroid being 20 mg. The relationship between dosage and the risk of complications was not further explored. It can be related since most of the steroid users received steroid dosage of >17.5 mg, and only 7.3% of the study participants received steroids of less than 17.5 mg. These findings are particularly relevant in the ICU setting, where patients are already at increased baseline risk for infection due to comorbidities, invasive devices, and antibiotic exposure.

Minderhoud et al. conducted a study in which HSV-1 reactivation was noted after a cumulative dosage of 420 mg of prednisolone over four weeks. In this case, the patient received a cumulative dose of approximately 225 mg of prednisolone equivalent (equivalent to 900 mg of hydrocortisone) over four days, which is lower than the dose mentioned in the study [4]. Herpes simplex virus type 1 (HSV-1), a neurotropic virus that establishes latency in the trigeminal ganglion, may reactivate under physiologic stress or immunosuppressive states [5]. Acute illness, coupled with corticosteroid use, likely led to the reactivation of herpes simplex virus in this patient. Despite not using viral PCR for confirmation, the dermatomal distribution (V2) and mucosal involvement were clinically consistent with HSV-1 dermatitis and gingivostomatitis, and the patient responded well to antiviral therapy.

In this case, the subsequent development of *Candida glabrata* fungemia further supports the profound, though transient, immunosuppressive effect of short-term steroids. *Candida glabrata* is a non-albicans *Candida* species that has become a significant cause of invasive candidiasis in patients in intensive care units (ICUs). It is particularly concerning due to its intrinsic and acquired resistance to azole antifungals. Risk factors include central venous catheters, broad-spectrum antibiotic use, parenteral nutrition, and, critically, immunosuppressive therapy [6]. Although no central lines were placed, our patient had a Foley catheter placed on day 2 of admission, which could have been a source of infection. Experimental studies have demonstrated that glucocorticoids inhibit macrophage autophagy and impair reactive oxygen species production, key mechanisms by which macrophages clear fungal pathogens, such as *C. glabrata*. These alterations may facilitate fungal translocation from the gastrointestinal tract or persistence in the bloodstream. A recent meta-analysis by Li and Denning reported a 213% increase in mortality associated with corticosteroid use in patients with candidemia, highlighting the severity of this complication [7,8]. The dual infection with HSV-1 and *Candida glabrata* is particularly interesting, as animal models have shown that HSV-1-infected macrophages are inhibited in their anti-*Candida* function [9]. While echinocandins are considered first-line therapy for invasive *C. glabrata* infections due to their fungicidal activity and favorable resistance profile, the optimal management strategy includes early source control, the removal of central lines and Foley catheters when possible, and daily blood cultures until clearance [6,10].

In our patient, the combination of brief corticosteroid exposure, type 2 diabetes mellitus, antibiotic therapy, and a compromised physiologic state created a susceptible environment for dual opportunistic infections, HSV-1 and *C. glabrata*. This underscores the need for a careful risk-benefit assessment when initiating corticosteroids, even in guideline-supported indications such as septic shock. It also emphasizes the importance of close monitoring for secondary infections and the necessity of source control, particularly in patients with pre-existing comorbidities such as diabetes and chronic kidney disease or prior urologic procedures.

## Conclusions

This case underscores the significant yet often underestimated risk of opportunistic infections, including HSV-1 reactivation and *Candida glabrata* fungemia, associated with even brief courses of corticosteroid therapy in critically ill patients. While systemic corticosteroids are indispensable in managing vasopressor-refractory septic shock, their immunomodulatory effects may transiently impair host defenses, creating susceptibility to severe infectious complications. Clinicians must balance the therapeutic benefits of corticosteroids against potential risks, maintaining heightened vigilance and promptly investigating new clinical symptoms suggestive of opportunistic pathogens. Early identification, source control, rapid initiation of appropriate antifungal and antiviral treatments, and careful consideration of predisposing comorbidities remain essential to optimizing outcomes in this vulnerable patient population. This case adds to the growing body of literature suggesting that steroid-related infectious risks are not confined to long-term use and may occur acutely in vulnerable patients. Future research should further investigate the nuances of corticosteroid-induced immunosuppression and the relationship of dosage with the risk of infections to refine guidelines and improve outcomes in this high-risk patient population.

## References

[1] Evans L, Rhodes A, Alhazzani W (2021). Surviving sepsis campaign: international guidelines for management of sepsis and septic shock 2021. Intensive Care Med.

[2] Cain DW, Cidlowski JA (2017). Immune regulation by glucocorticoids. Nat Rev Immunol.

[3] Waljee AK, Rogers MA, Lin P (2017). Short term use of oral corticosteroids and related harms among adults in the United States: population based cohort study. BMJ.

[4] Minderhoud TC, van Meer MP, van Thiel RJ, den Hoed CM, van Daele PL, Schurink CA (2018). [Infections during glucocorticoid use] (Article in Dutch). Ned Tijdschr Geneeskd.

[5] Saleh D, Yarrarapu SN, Sharma S (2023). Herpes simplex type 1. StatPearls [Internet].

[6] Fidel PL Jr, Vazquez JA, Sobel JD (1999). Candida glabrata: review of epidemiology, pathogenesis, and clinical disease with comparison to C. albicans. Clin Microbiol Rev.

[7] Yang Z, Wang X, Dong T, Zhao WJ, Li H (2024). Impact of glucocorticoids and rapamycin on autophagy in Candida glabrata-infected macrophages from BALB/c mice. Front Immunol.

[8] Li Z, Denning DW (2023). The impact of corticosteroids on the outcome of fungal disease: a systematic review and meta-analysis. Curr Fungal Infect Rep.

[9] Cermelli C, Orsi CF, Ardizzoni A, Lugli E, Cenacchi V, Cossarizza A, Blasi E (2008). Herpes simplex virus type 1 dysregulates anti-fungal defenses preventing monocyte activation and downregulating toll-like receptor-2. Microbiol Immunol.

[10] Pappas PG, Kauffman CA, Andes DR (2016). Clinical practice guideline for the management of candidiasis: 2016 update by the Infectious Diseases Society of America. Clin Infect Dis.

